# Virulence profile: Mike Kelso

**DOI:** 10.1080/21505594.2017.1380962

**Published:** 2017-11-24

**Authors:** Mike Kelso

**Affiliations:** Illawarra Health and Medical Research Institute and School of Chemistry, University of Wollongong, Wollongong, NSW, Australia

## Tell us about your early days

I grew up in the suburbs of Brisbane on the east coast of Australia and completed my secondary education at St Joseph's College Gregory Terrace, an inner-city catholic (Christian Brothers) boy's school. Not really knowing what took my fancy career-wise, I naively entered university straight out of high school in 1989 to study engineering. It only took 3 weeks to realise that I would never cut it as an engineer, so I undertook a ‘gap’ year. Following that, it took two years of studying pharmacy to convince me that I didn't want to be a pharmacist and that I instead had a passion for chemistry and drug discovery. This triggered a move to the University of Wollongong (UOW) where in 1996 I was part of the first cohort of graduates from the newly badged B. Medicinal Chemistry (Hons) degree, the first of its kind in Australia.

## Tell us about your education and experiences at university

During my PhD studies I became interested in how to stabilize peptides into α-helical conformations. I was part of a team who discovered that clipping together histidine residues spaced 4 amino acids apart with a metal ion (Pd^2+^) imparts substantial α-helical character into otherwise unstructured peptides. These innovative discoveries were published in great chemistry journals and helped me to secure a prestigious postdoctoral fellowship from the Australian National Health and Medical Research Council (NHMRC). Before undertaking my fellowship I moved to San Sebastian in the Spanish Basque Country for one year (2003) to advance my synthetic chemistry skills in catalytic asymmetric synthesis. With some hard work and a dash of good fortune my team discovered an efficient new reaction (aza-Michael addition of carbamates to α,β-unsaturated enones) for the stereoselective synthesis of β-amino acids.

The outward two-year overseas leg of my NHMRC CJ Martin Fellowship (2004–2005) was spent at The Scripps Research Institute with Prof Dale Boger, where I worked on an academic/industry collaboration with Johnson and Johnson Pharmaceuticals exploring Fatty Acid Amide Hydrolase inhibitors for use in pain. While not fruitful in terms of academic outputs, this period was instrumental in securing fundamental knowledge of modern drug discovery and development (i.e. structure-activity relationships, pharmacokinetics, safety pharmacology etc.). Energised by an amazing experience as a Scripps postdoc I returned to my alma mater UOW for the inward two-year leg of my fellowship (2006–2007) keen to apply my newly acquired medicinal chemistry knowledge. Under the distinguished tutelage of Prof John Bremner my focus moved towards the rational design and synthesis of antibacterials acting via novel mechanisms; a theme that persists to this day in my research.

## What was your first position after university?

The timely retirement of Prof Bremner in 2007 created an opening for me at UOW and in 2008 I was appointed as a lecturer in chemistry. With a modest start-up package (but fully fitted out lab!) I commenced my independent academic career with gusto, looking to make my mark in anti-infectives. A decade later, and looking back as an Associate Professor, I can't help but feel proud of my groups achievements.

## What areas or topics does your lab currently focus on?

Examples of discoveries from the lab include low molecular weight pyrazine-based antibacterials against MRSA, *Clostridium difficile*-selective antibacterials, efflux-pump inhibitor-antibacterial hybrid drugs, efflux-pump inhibitor-photosensitiser hybrids for use in antimicrobial photodynamic therapy and nitric oxide-donor cephalosporins for dispersing bacterial biofilms. I was awarded the prestigious Royal Australian Chemical Institute's (RACI) 2013 National Medicinal Chemistry (Biota) Prize for the cephalosporin discoveries. My group is also actively investigating inhibitors of the bacterial sliding clamp and an exciting new class of compounds that shows remarkable activity against *Mycobacterium tuberculosis*. My research has a translational flavour with patenting and pursuit of commercial partnerships key considerations. The only work done in my lab is synthetic chemistry so we draw heavily on partnerships with expert local, national and international collaborators. Some of these include Nick Dixon and Aaron Oakley (UOW), Staffan Kjelleberg and Scott Rice (NTU, Singapore), Jeremy Webb and Saul Faust (University of Southampton, UK), George Tegos and Mike Hamblin (Harvard Medical School, USA), Eleftherios Mylonakis (Brown University, USA) and Gregory Cook (University of Otago, NZ).

## What are your research interests and your philosophy in scientific pursuits?

My ultimate goal is to create innovative and clinically useful anti-infectives that act via novel mechanisms. Given the ominous approach of the ‘post-antibiotic era,’ I believe it is the responsibility of academic medicinal chemists like myself to enable the antibiotic solutions of the 21^st^ century.

## What do you do for fun?

Surfing is a big part of my life and indeed it played a major role in influencing where I looked for postdoctoral opportunities. Having the good fortune to live and work in the Basque Country and San Diego and to eventually settle in Wollongong, all well known for the quality of their surf breaks, was not an accident. Identifying a passion in life above and beyond your career, and finding a way to bring them together, is a great path to happiness and success!

